# Sinonasal Tissue Remodelling during Chronic Rhinosinusitis

**DOI:** 10.1155/2021/7428955

**Published:** 2021-09-16

**Authors:** Satya Amirapu, Kristi Biswas, Fiona J. Radcliff, Brett Wagner Mackenzie, Stephen Ball, Richard G. Douglas

**Affiliations:** ^1^Department of Surgery, The University of Auckland, Auckland 2043, New Zealand; ^2^Department of Molecular Medicine and Pathology, The University of Auckland, Auckland 2043, New Zealand

## Abstract

The purpose of this review is to summarise contemporary knowledge of sinonasal tissue remodelling during chronic rhinosinusitis (CRS), a chronic disease involving long-term inflammation of the paranasal sinuses and nasal passage. The concept of tissue remodelling has significant clinical relevance because of its potential to cause irreversibility in chronic airway tissues. Recent studies have indicated that early surgical treatment of CRS may improve clinical outcome. Tissue remodelling has been described in the literature extensively with no consensus on how remodelling is defined. This review describes various factors implicated in establishing remodelling in sinonasal tissues with a special mention of asthma as a comorbid condition. Some of the main histological features of remodelling include basement membrane thickening and collagen modulation. This may be an avenue of research with regard to targeted therapy against remodelling in CRS.

## 1. Introduction

Epidemiological data suggest chronic rhinosinusitis (CRS) affects at least 5% of the general population [1]. Despite protocolised medical and surgical intervention from international consensus documents recurrence rates remain problematic [2]. The aetiology of the recurrent disease remains elusive. Recent data suggest early surgical intervention may reverse disease progression and prevent recurrence [3]. Research indicates that patients who receive surgery within 12 months of being diagnosed with CRS have a significant reduction in healthcare compared with those patients treated after more than five years of duration of disease [4].

The respiratory mucosa of the nasal passage is continuously exposed to the particles in the inhaled air. In health, the epithelium, cilia, and mucus function together to ensure efficient mucociliary clearance. Impaired mucociliary clearance in CRS likely represents one of the first pathogenic steps in the chronic nature of this disease [5]. The sinus ostia in patients with CRS are obstructed and altered microbiota profiles and mucosal inflammatory responses are observed [5]. Tissue remodelling is a key element in the resultant histopathological changes of the mucosa.

**Figure 1:**
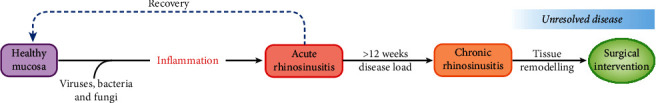
Progression of disease in chronic rhinosinusitis. Inflammation observed in acute rhinosinusitis caused by viruses, bacteria, and fungi can resolve to a healthy state or can proceed to CRS if the inflammation persists longer than 12 weeks. The chronic nature of disease induces widespread tissue remodelling of the paranasal sinuses and nasal passage. Unresolved CRS often requires surgical intervention to remove purulent, stagnant mucous, and inflamed tissue.

Tissue remodelling comprises the cyclical deposition and degradation of extracellular matrix (ECM) [6, 7]. This dynamic process is an integral part of the tissue response to injury or inflammation in general. Tissue remodelling often leads to a restoration of normality; however, an aberration in this process results in progression to a pathological state [8] (Figure 1).

Traditionally, tissue remodelling is viewed as a secondary phenomenon, appearing late in the disease process consequent to persistent inflammation [9]. However, more recently tissue remodelling after an injury has been shown to occur in parallel to inflammation, rather than the two being consecutive phases of the response to injury [10–12]. These observations suggest remodelling may occur early in the disease process in CRS. Pathological endotyping or subphenotyping of remodelling features may potentially identify patients at higher risk of recurrent or persistent disease.

**Figure 2:**
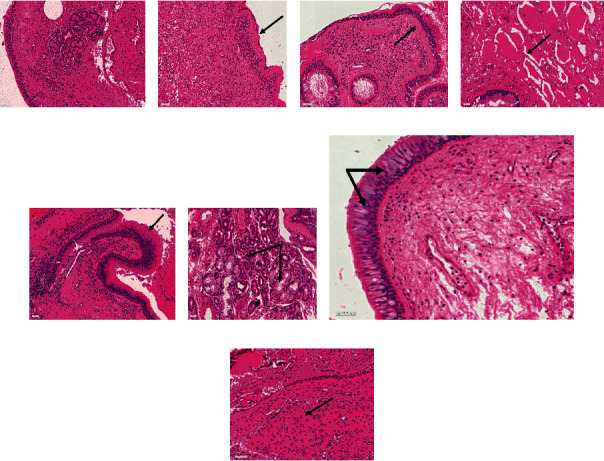
Key histological features of sinonasal mucosa remodelling during chronic rhinosinusitis (CRS). The haematoxylin and eosin-stained sections display tissue remodelling in sinonasal tissues from patients with CRS. (a) Normal mucosa, (b) epithelial denudation, (c) basement membrane thickening, (d) oedema, (e) epithelial hyperplasia, (f) glandular hyperplasia, (g) goblet cell hyperplasia, and (h) fibrosis. All images are captured at 20.05 x magnification. Black arrows indicate the different regions of interest.

There is currently no consensus on how to define remodelling. The most common approach is to identify squamous metaplasia, subepithelial fibrosis, and basement membrane thickening [13]. This implies tissue remodelling in the sinonasal mucosa involves both the epithelium and interstitium (subepithelium or lamina propria) (Figure 2). Other factors may also contribute to tissue remodelling. The nature of the inflammatory and remodelling responses associated with CRS is likely to be determined by genetic and epigenetic factors [14].

Currently, CRS is considered a heterogeneous disease, with numerous predisposing factors with similar symptomatology, making it challenging to categorise CRS into clinically relevant subtypes [15]. They are traditionally classified into specific, clinical phenotypes based on the presence (CRSwNP) or the absence of nasal polyps (CRSsNP) [1]. Histologically, CRSwNP shows a predominance of interstitial oedema, whereas fibrotic remodelling of the lamina propria is the hallmark of CRSsNP [18], but there is considerable overlap in both clinical and histological presentation of disease [17]. Additional endotypic or histological phenotypes based on the presence or absence of eosinophils and cytokines are currently being investigated [16]. In this review, we will discuss only idiopathic CRS and local sinonasal inflammation where surgery can be performed.

Identification and classification of the histological features of remodelling may provide insights into why some patients with CRS do not respond to therapy, particularly those with severe asthma. One possibility is that remodelling causes irreversible damage to respiratory mucosa [19].

In this review, we summarise the current knowledge of remodelling in idiopathic CRS. The association of ECM, stromal cells, cytokines, the influence of thrombin, sinonasal inflammation, and the sinus microbiome on tissue remodelling are discussed. Moreover, the clinical implications of tissue remodelling in CRS to prognosis and response to treatment will be presented, particularly in the light of the evidence that suggests that early treatment may improve outcome.

## 2. The Role of Extracellular Matrix in Remodelling

The ECM is essential for maintaining tissue function in the presence of ongoing minor trauma or inflammation [20]. The ECM is composed of several hundred proteins, embedded in an amorphous gel-like substance made of proteoglycan [21] which allows cells to attach to the ECM. The proteoglycans are negatively charged due to the presence of sulphated glycosaminoglycans [22]. Proteoglycans occupy a relatively large volume within the tissues and interact with positively charged molecules like cytokines and growth factors, causing the local accumulation of these molecules [23].

There are two main components of the ECM in sinus tissue: the interstitial connective tissue and the basement membrane. Collagen (types I–III, V, and XI) is a structural protein of the interstitial connective tissue that scaffolds the sinonasal submucosa. Proteoglycans (PG) such as versican, decorin, and aggrecans are macromolecules interspersed along collagen fibrils. In contrast, the basement membrane contains denser collagen (type IV), along with laminins, heparan sulphate proteoglycans (HSPGs), nidogen, and entactin [24].

The ECM is known to influence immune cell behaviour in inflamed tissues and can modulate immune function. In chronically inflamed tissue, irregularities caused by ECM altered by tissue remodelling processes can contribute to the altered immunological state of the tissues [25]. Eosinophils and collagens I, III, and V deposition in the ECM and TGF-*β*-1 can be detected in the mucosa of CRS tissues. The accumulation of eosinophils can potentially damage tissue and may be an essential driver of tissue remodelling. An increase in eosinophils is observed in the sinonasal tissues of patients with CRSwNP. This increase in eosinophils encourages the production and deposition of collagen in the ECM [8, 26]. The basement membrane (BM) thickening in CRS by secretion of TGF-*β* by eosinophils results in activation of fibroblasts and increased production of ECM proteins [27].

In the last decade biomarkers of ECM that play a role in remodelling in CRS, such as periostin (POSTN) and osteopontin (OPN), have been described. POSTN and OPN are matricellular proteins with cell-matrix interaction functions [28]. The first description of POSTN in sinonasal mucosa indicated that it was expressed in normal mucosa and significantly overexpressed in patients with CRSwNP [29]. A reduction in the expression of POSTN was observed following successful functional endoscopic sinus surgery (FESS), suggesting POSTN is a possible CRS biomarker that correlates with postoperative disease resolution [30].

OPN is also associated with wound repair and fibrosis generally [28]. OPN expression is markedly upregulated in CRSsNP and CRSwNP tissues compared to inferior turbinate tissues from patients without CRS disease. OPN upregulation is also detected in patients with asthma [31]. Moreover, in CRS, OPN is expressed in the nasal epithelium, submucosal glands, and the ECM, suggesting a role in tissue remodelling and ECM turnover. These ECM proteins, particularly POSTN, are associated with the increased severity of CRSwNP [32]. What remains unclear, however, is how early, in CRS pathogenesis, POSTN and OPN overexpression occurs and how these influence subsequent tissue remodelling in late-stage CRS.

## 3. Role of Cytokines in Remodelling

**Figure 3:**
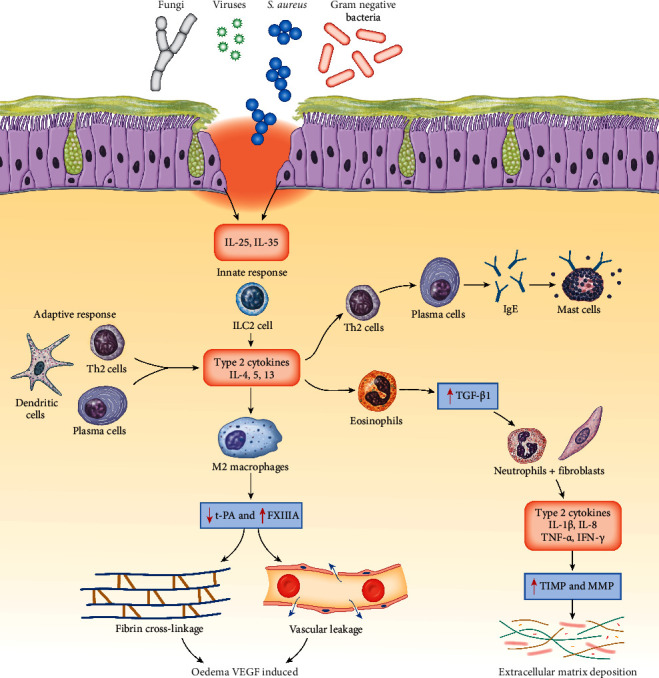
Cytokine expression in the pathogenesis of remodelling in chronic rhinosinusitis. Epithelial barrier disruption induces inflammation, initiating adaptive and innate immune response which creates a cascade of cytokines. This leads to fibrin deposition through coagulation factors like thrombin and Factor XIIIA, oedema through VEGF, and extracellular matrix deposition through TGF-*β*1. VEGF = vascular endothelial growth factor, TGF-*β*1 = transforming growth factor-*β*1, IL-1*β* = interleukin-1*β*, IL-8 = interleukin-8, TNF-a = tumor necrosis factor-a, IFN-*γ* = interferon gamma, TIMP-1 = tissue inhibitor of metalloproteinase-1, MMP = metalloproteinases, and tPA = plasminogen activator.

Cytokine expression and regulation are considered critical factors in the pathogenesis of CRS [33] (Figure 3). A distinctive pattern of cytokine production is observed during the remodelling of inflamed tissue [34]. Research has shown that some of these cytokines, like transforming growth factor (TGF-*β*1), are highly expressed in the early stages of CRSwNP [11].

Dysregulation of TGF-*β*1 is associated with increased differentiation of fibroblasts into myofibroblasts, which deposit ECM [35]. The TGF-*β*1 dimer is generated intracellularly and stored in an inactive form in the cytoplasm. This dimer needs to be activated by latency appreciated peptide (LAP) and latent TGF-*β*1-binding protein (LTBP-1) [36]. Integrins then activate TGF-*β*1, whose expression is increased in CRS [35, 37]. Furthermore, the differentiation of T cells to regulatory T (Treg) cells by TGF-*β*1 may lead to the specific inflammatory patterns seen in CRS [38].

TGF-*β*1 accumulates in the ECM and stroma [39]. An increase in interstitial collagen causes fibrosis and contributes to BM thickening, both of which are characteristics of CRSsNP [40]. Contrastingly, TGF-*α* may have a role in angiogenesis, contributing to the stroma observed in CRSwNP, and with the recruitment of inflammatory cells, downstream effects such as increased epithelial proliferation and squamous metaplasia are seen [41]. Additionally, 50% of eosinophils in nasal polyps have been identified as positive for TGF-*β*1 [42]. The involvement of TGF-*β*1 in remodelling and inflammation makes this cytokine a potential target for therapy [6, 35].

The matrix metalloproteinases (MMPs), fibroblast growth factor (FGF), and bone morphogenetic factors (BMP) are all known regulators of tissue remodelling [33]. MMPs are a family of zinc-dependent and calcium-dependent endopeptidases [43] which are produced by the respiratory epithelial cells, mast cells, neutrophils, and fibroblasts [44]. MMPs play a crucial role in the breakdown of basement membranes and ECM proteins, including collagen types IV, V, VII, X, XIV, gelatin, and elastin contributing to oedema and remodelling [44]. Increased expression of MMP-9 and their regulators, tissue inhibitors of metalloproteinases (TIMPs) [40], have been found in patients with recurrent CRS [41]. Furthermore, a high TIMP-1/MMP-8 ratio is associated with an improved outcome after surgical treatment in CRSwNP, suggesting a role in remodelling [45]. This ratio correlates with disease severity and may predict a lack of response to oral corticosteroids associated with asthma [46], but this remains to be demonstrated in CRS. Metalloproteinases implicated in CRS may serve as a target for novel therapies and for evaluating prognosis [45].

Vascular endothelial growth factor (VEGF) is another cytokine that plays a critical role in vascular remodelling, causing vascular permeability and accumulation of ECM proteins [47]. Vascular endothelial cells, fibroblasts, smooth muscle cells, macrophages, and white blood cells secrete VEGF and may contribute to the formation of oedema in polyps [48]. Prostaglandin D2(PGD2) promotes VEGF release from NP-derived fibroblasts (NPDF) via the D-prostanoid receptor and further promotes oedema formation and angiogenesis [49].

To summarise, numerous inflammatory mediators have been implicated in the mechanism of development of nasal polyps. However, a precise mechanism of why recurrence of polyps occurs in CRSwNP associated with asthma is not clear [47]. Perhaps, the cellular characteristics of remodelling may elucidate what contribute to early and late remodelling in CRS tissues.

## 4. Role of Inflammatory and Stromal Cells in Remodelling

Chronic inflammation may be induced by stromal cells activating immune cells [50]. Mesenchymal-derived cells such as the fibroblasts are the most abundant stromal cells and are implicated in the overproduction of ECM [51]. Under normal physiological conditions, fibroblasts produce the ECM constituents such as type I, III, and V collagen and fibronectin. They produce MMPs in the ECM and basement membrane comprised of collagen IV and laminin [52].

The early development of nasal polyps depends on the conversion of fibroblasts into myofibroblasts whose activity is expressed by *α*-smooth muscle actin (*α*-SMA) and overproduction of ECM proteins including POSTN [8]. Myofibroblasts, in turn, modulate the different components of the ECM, like the proteoglycans, elastin, and collagen, including matricellular proteins like POSTN [53]. POSTN is secreted in response to interleukins IL-4 and IL-13 [54] and is involved with subepithelial fibrosis through interactions with integrins [55].

Macrophages play a valuable role in tissue remodelling after damage through phagocytosis of damaged cells. In particular, activated macrophages (M2 type) reduce the inflammatory response and initiate angiogenesis and tissue remodelling [56, 57]. In CRS, subepithelial eosinophils and M2 type macrophages are increased significantly in early-stage nasal polyps when compared to mature polyps and normal mucosa [11].

## 5. Role of Coagulation Factors in Remodelling

Coagulation and fibrin formation occur early in response to injury. The role of the coagulation system in tissue remodelling has been examined with a particular reference to thrombin [58]. During activation of the coagulation system, thrombin promotes remodelling by secretion of VEGF from the respiratory epithelial cells [59]. Usually, airway inflammation increases vascular permeability and plasma coagulation factors exude into the extracellular space [60]. Thrombin can further stimulate the release of cytokines such as IL-6, PGE2, PDGF, and MUC5, which encourages eosinophil migration and airway permeability.

Recent studies in CRSwNP have reported excessive fibrin deposition as a result of decreased fibrinolytic activity and increased coagulation factors [61], activating thrombin [62]. Thrombin activation lowers tissue plasminogen activator (tPA) levels and upregulates Factor XIII (FXIIIA) [58]. This overproduction of FXIIIA by M2 macrophages is thought to cause remodelling of nasal polyps via fibrin deposition, plasma protein retention, and angiogenesis [61]. Moreover, thrombin-induced VEGF may be involved in the formation of recurrent nasal polyps in CRSwNP with asthma [62].

## 6. *Staphylococcus aureus* May Influence Remodelling in CRS

There is some evidence linking colonisation of the potentially pathogenic bacterium *Staphylococcus aureus* to remodelling in CRSwNP. This bacterium degrades the epithelial barrier and drives inflammation via the production of endotoxins that act as superantigens to generate a T-cell-mediated inflammatory cascade [63, 64]. Immunoglobulin *E* (IgE) produced by immune cells in response to staphylococcal endotoxins also has a direct effect on eosinophils [65]. These findings correlate with increased levels of IL-5 and eosinophilic cationic protein (ECP) detected in nasal polyps caused by *S. aureus* that result in remodelling [63, 66].

**Figure 4:**
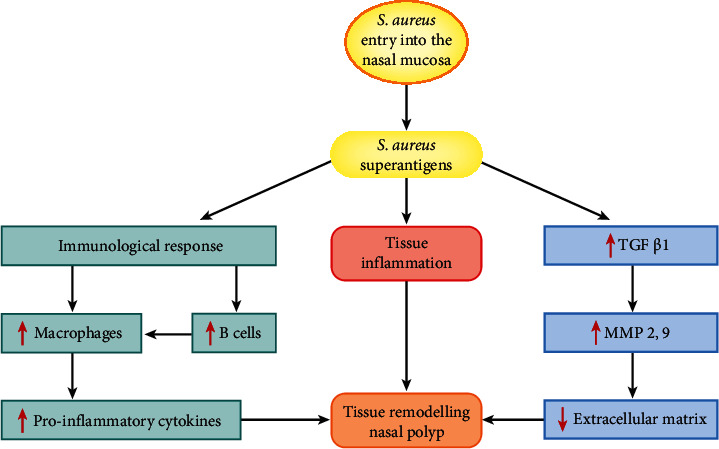
S*taphylococcus aureus* influences tissue remodelling in patients with chronic rhinosinusitis and nasal polyps. *S. aureus* drives inflammation via the production of endotoxins that act as superantigens to generate a T- and B-cell-mediated cascade. Moreover, through TGF-*β* and MMP-9 and MMP-2, the extracellular matrix may be laid down inducing remodelling. TGF-*β*1 = transforming growth factor-*β*1 and MMP 2,9 = metalloproteinases 2,9.

The lack of TGF-*β* expression in nasal polyps combined with increased expression of IgE promotes mast cell degranulation and subsequent attraction of eosinophils to the site of inflammation. The lack of expression suggests decreased T-cell function [67]. Moreover, TGF-*β* plays a role in the ECM metabolism by stimulating the production of TIMP-1, inhibiting the enzymatic breakdown of the ECM, and inducing a remodelled tissue in CRSwNP. The increased cysteinyl leukotriene production by mast cells further worsens the Th2-mediated inflammation [63]. Therefore, the combination of innate and acquired immunological mechanisms explains how the remodelling and an immune response occur together in the presence of *S. aureus* (Figure 4).

The persistence of *S. aureus* is also hypothesized to play a role as a potential inducer of chronicity in nasal polyps as it can reside within fibroblasts. In CRS, a dysfunctional immune barrier characterised by inflamed mucosal epithelium, atopic conditions, and obstructive sinuses may provide a platform for secondary bacterial infection and dysbiosis, leading to chronic inflammation [68]. Moreover, specific IgE antibodies to *Staphylococcus* enterotoxins have been associated with a more severe inflammatory response in nasal polyps, particularly with comorbid asthma [69]. Treating patients early with surgery may help restrict the progression and recurrence of nasal polyps as well as controlling comorbid asthma.

## 7. Clinical Implications of Tissue Remodelling in CRS

Tissue remodelling in CRS influences the chronicity and recalcitrance of disease and may be associated with comorbid asthma [9]. However, whether this tissue remodelling is reversible [9] and what impact the resulting features have on the quality-of-life indicators [70] remain unclear.

Functional endoscopic sinus surgery (FESS) generally improves clinical outcomes and remains the mainstay of treatment of CRS cases that do not respond to medical therapy [13]. It has been suggested that delaying surgical treatment may allow the disease to progress to an irreversible state [3]. A delay in FESS is associated with higher rates of asthma [71]. There is also emerging evidence to show that repeated surgical intervention may lead to more severe tissue and bone remodelling [72].

We have focused on the use of surgery to correct anatomical obstructions, but there are many systemic diseases associated with CRS where surgery may not be appropriate. For example, systemic diseases like aspirin-exacerbated respiratory disease can be the cause of or alter the prognosis of CRS, contributing to the heterogeneity that exists currently [73]. Although patients with aspirin sensitivity are medically managed with salicylates, anti-leukotriene agents, and aspirin desensitisation, recent evidence shows that sinus surgery decreases aspirin-induced reaction severity [74].

Systemic allergy can contribute to inflammation in CRS and nonsurgical treatment may ameliorate any contribution to the overall sinonasal inflammation in a CRS patient [16, 73]. Biologics like omalizumab, an anti-immunoglobulin E (anti-IgE) monoclonal antibody, may be effective in treating CRSwNP with comorbid asthma as this systemic therapy might be useful to simultaneously control upper and lower respiratory disease [75]. Targeting key mediators of allergic inflammation such as IgE, IL-5, and IL-4/IL-13 constitutes a novel therapy in patients suffering from CRSwNP and their applicability to patients with CRSwNP refractory to standard treatment is underway [76].

The role of corticosteroids in CRS tissue remodelling is debatable. Previous research shows that the effect of corticosteroid therapy may not reduce collagen content in the basement membrane. More recently, corticosteroid therapy is thought to reverse the process of remodelling by reducing the collagen content in the basement membrane [9, 77].

Aberrant tissue remodelling has been shown to adversely affect mucociliary function [70], resulting in prolonged duration of CRS symptoms in those patients with asthma and aspirin sensitivity [27]. Evidence-based research in the lower airways may indicate that an increase in basement membrane thickness may be protective [78]. However, this finding has not been reported in the upper respiratory tract. Modulation of basement membrane may be an avenue of research with regard to targeted therapy against remodelling in CRS, based on research indicating the thickness of the basement membrane may correlate with the duration of disease [27].

## 8. Conclusions

The published literature suggests tissue remodelling occurs in CRS and distinct histological features can be used to differentiate the current phenotypes of CRS. Various inflammatory processes contribute to persistent tissue remodelling in CRS. It remains unclear whether such remodelling is irreversible without surgical intervention or whether improved clinical outcomes after treatment are reflected at the tissue level. Investigation at a histological level aimed at controlling or reversing CRS-associated remodelling early in the disease process is warranted to enable better prediction of clinical outcomes.
